# Emergency deep hypothermic circulatory arrest in a 1-year-old undergoing cardiac surgery in a Nigerian hospital - anaesthesia and critical care interventions

**DOI:** 10.11604/pamj.2020.37.221.26557

**Published:** 2020-11-05

**Authors:** Chidiebele Samuel Ikenga, Balswaroop Sahu, Vincent Okwulehie, Friday Umeh

**Affiliations:** 1 Anaesthesia Department, University of Nigeria Teaching Hospital, Ituku Ozalla, Enugu, Nigeria,; 2 Sri Satya Sai Sanjeevani Hospital, Nava Raipur, Atal Nagar, Chattisgarh, India,; 3 Surgery Department, University of Nigeria teaching Hospital, Ituku Ozalla, Enugu, Nigeria

**Keywords:** Deep hypothermic circulatory arrest, cerebral perfusion, interrupted aortic arch, aberrant right subclavian artery, ventricular septal defect, patent ductus arteriosus

## Abstract

Deep Hypothermic Circulatory Arrest (DHCA) is a technique used to obtain optimal operating conditions while providing cerebral protection. The case report presented a DHCA on an infant that was basically done as an emergency in an attempt to correct a previously unrecognized anomaly. We report a case of a 1-year-old that had surgery for Ventricular Septal Defect (VSD) and Patent Ductus Arteriosus (PDA) ligation but following closure was noted to have no palpable peripheral pulses, only carotid pulsation. This necessitated an emergency reopening of the chest. Close inspection revealed an interrupted aortic arch with aberrant right subclavian artery and pre-surgery PDA supplying both upper and lower limb. DHCA was immediately commenced and the patient cooled to 16°C. The surgeon promptly set out to attach the subclavian artery to the ascending aorta and descending aorta. At the completion of the surgery, the patient was taken to Intensive Care Unit (ICU) for critical care support. She was subsequently discharged after spending a little more than a week in ICU. This procedure is rarely done as an emergency but was instituted in our case in effort to immediately achieve perfusion to the limbs. As the expertise to carry out the procedure is limited, it might be better to develop ways to efficiently ensure the skill set is continually updated.

## Introduction

Deep hypothermic circulatory arrest is a technique used to obtain optimal operating conditions while providing cerebral protection. Most cardiac surgical procedures can be accompanied using cardioplegia-induced cardiac arrest and cardiopulmonary bypass to maintain perfusion. In some situations, however, the underlying pathology or the nature of the surgery proposed necessitates complete cessation of the circulation. The use of profound systemic hypothermia to preserve organ function during cessation of circulation is termed deep hypothermic circulatory arrest [1]. In our case, DHCA was unplanned and followed almost immediately after surgery for VSD repair and PDA ligation.

## Patient and observation

We present a 1-year-old female was admitted for VSD repair and PDA ligation. Recently the patient was suffering from fever and coughs underwent treatment and completely recovered. There is marked failure to thrive with child weighing only 5kgs and height of 70cm. Heart rate at presentation was 140b/m, RR of 38 cycles/min and Bp of 80/50mmhg. On auscultation, S1 and S2 were heard with pansystolic murmur and splitting of S2. There were coarse crepitations bilaterally on auscultation of the chest. The patient was conscious and alert, OFC-40cm, pupil equal and bilaterally reactive, normal tone globally, power 5 on all limbs. An assessment of failute to trive (FTT) with acyanotic heart disease and right bronchopnemonia (treated) was made. An echocardiography was made with a diagnosis of Ventricular Septal Defect (VSD) and Patent Ductus Arteriosus (PDA) and patient was scheduled for surgery for VSD closure and PDA ligation. Other investigations FBC, serum electrolytes, urea and creatinine were all within normal ranges. An American Society of Anaesthesiology (ASA) grade of 2 was made and patient prepared for surgery the next day. She was fasted from 12 midnight for solid foods and allowed for breast feeding till 3: 00 am. A peripheral line of 24G was secured the morning of the surgery. In the theatre, standard monitoring (electrocardiogram and pulse oximeter) was attached. The patient was sedated with ketamine 5mg and midazolam 0.5mg and manual ventilation started with 100% oxygen. An arterial line was inserted in the right radial artery with 24G canula for invasive blood pressure monitoring. The child was then intubated with 4.5 uncuffed tube facilitated by vecuronium 0.5mg and mechanically ventilation was started at tidal volume of 50mls/breath and respiratory rate adjusted to between 28-30 cycles/minutes. Intermittent fentanyl was used for analgesia as per requirement and isoflurane used for maintenance of anaesthesia. Central venous cannulation was inserted on the right internal jugular with triple lumen 4.5Fr. Surgery for VSD repair and PDA ligation started as per scheduled. After full heparinisation, cardiopulmonary bypass (CPB) was commenced, which lasted for 55mins. Total aortic cross clamp time was 55 minutes patient was successfully weaned off from CPB. ECG waveform was in normal sinus rhythm but invasive radial artery blood pressure monitoring showed dampened waveform. On palpation, normovolumic carotid artery pulsations on both sides were present equally. Proximal aortic arch palpation by surgeon was also normal. Protamine was given to reverse the effect of heparin.

**Figure 1:**
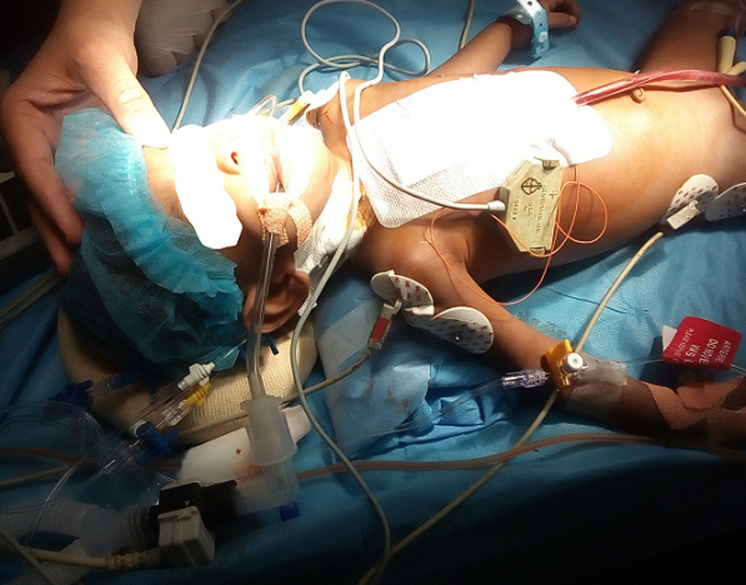
immediate post-operative picture before taking to ICU

**Figure 2:**
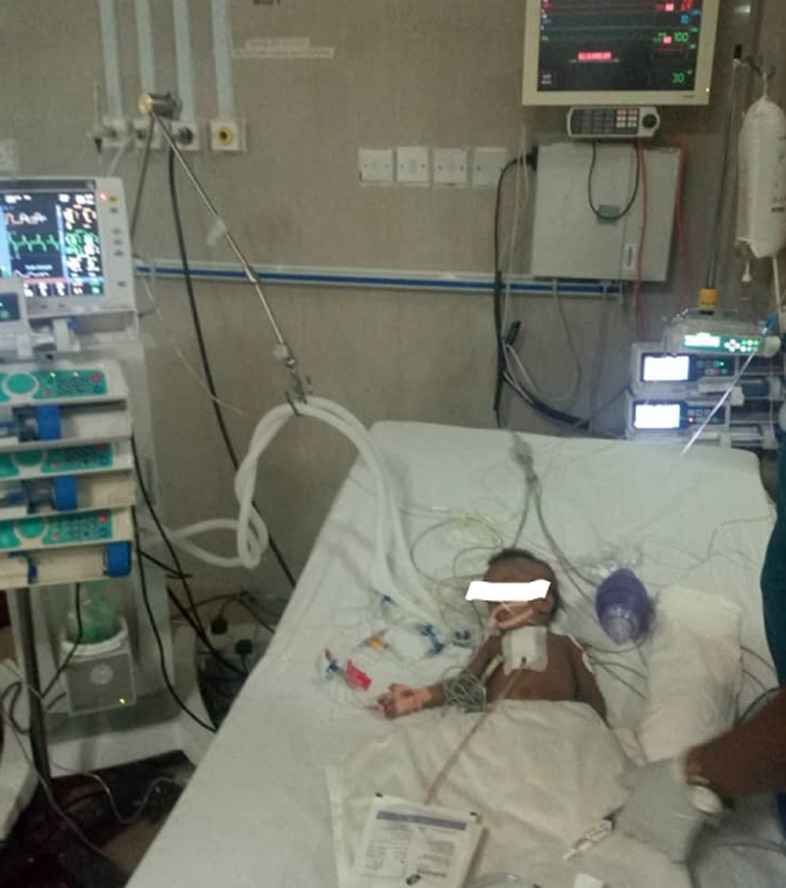
day 1 post-operative in ICU image

After chest closure, other arterial pulses were checked (left radial, femoral and dorsalis pedis) and revealed no pulsation. The peripheral extremities were also cold. The chest was immediately reopened after sternotomy. Inspection of the distal aortic arch by the surgeon revealed Interrupted aortic arch with aberrant right subclavian artery and PDA was supplying to both upper and lower limb. The patient was again placed on bypass and DHCA commenced gradually to a target of 16°C. Ice packs were also placed on the head of the patient. It took 20 minutes to achieve DHCA at 16°C and this (DHCA) lasted for 33 minutes while the subclavian artery was attached to the ascending aorta and descending aorta. The temperature was now gradually brought up to 35.7°C over 22 minutes. Heparin was reversed by Protamine. Adrenaline infusion (1.5mg/50mls) was started at 0.5mls/hour. Milrinone infusion (10mg/50mls) was started at 0.3mls/hr) and sildenafril (0.5mg/ml) started at 1.4mls/hr. Fresh frozen plasma and whole blood were transfused into the patient. Vital signs at end of surgery were BP- 79/37, HR-134, sp0_2_-100%, temperature at 35.2. Second surgery lasted 3 hours 10 minutes. At the end of the surgery in the theatre. The patient was transferred to ICU following surgery with endotracheal tube Insitu (Figure 1). In the ICU, combination of fentanyl and midazolam infusion were added to the drugs the patient was already on and stopped after 5 hours in the ICU. The Child was found to be have poor urine output and was placed on continuous furosemide infusion (20mg/20mls at 0.5 mls/hour. Intravenous paracetamol 75mg 8 hourly, pantoprazole 5mg 12 hourly, methyl prednisolone 50mg 12 hourly. The child was mechanically ventilated through the night. On 1 Day Post Operation (1DPO), meropenem was started for the child. Also, albumin was given because of low albumin. Milrinone was stopped and levosimendan (125mg/ml) commenced at 0.25mls/hr on 2DPO. Mgso4 and potasium chloride infusion were also given over some hours to correct hypomagnesemia and hypokalemia. The patient continued to be mechanically ventilated in ICU (Figure 2).

**Figure 3:**
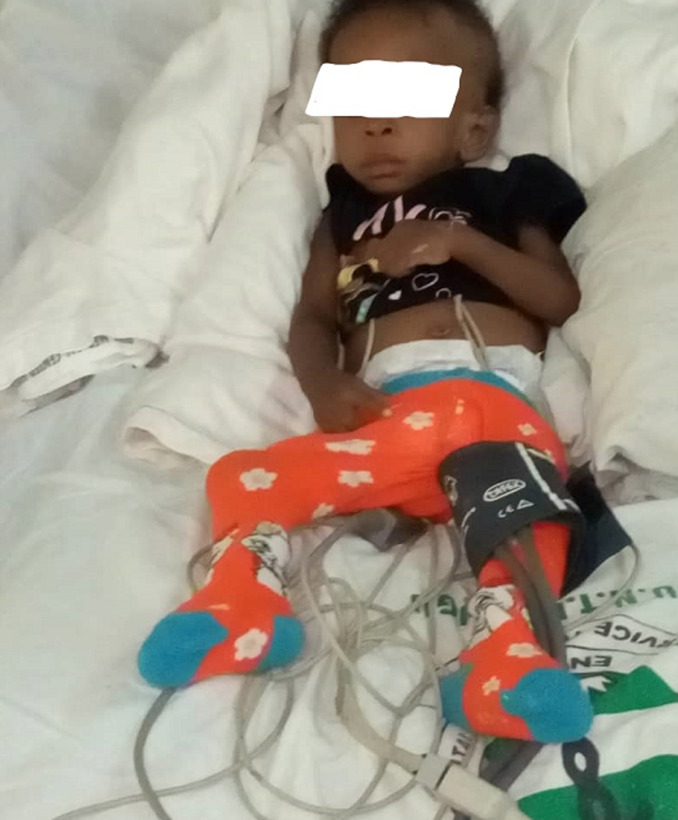
day 11 post-operative image, before discharge

On day 3 post OP, amino acid was commenced (astymin). Vital sign maintained on average of 75/55mmhg, sp0_2_100%, TDV 62mls, PEWEP 5, RR 30(still mechanically ventilated). Dexmedeto midine was commenced at 0.2mls/hour. Extubation of the child was done later in the afternoon and patient remained hemodynamically stable on intravenous fluid 4L/min of oxygen. Echocardiography done on the 4 day post op showed an expected performance of the heart. Oral rehydration salt (ORS) was commenced for the child. Sildenafil was commenced orally at 6.25mg 6 hourly at the same day. Aldactone was also commenced orally at 6.25mg 12 hourly 5 day post saw the commencement of oral feeds with adrenaline infusion tapered down and stopped. levosimendan and dexmedetomidine infusion were continued. IVF 5mls/hour was continued. There were also intravenous meronem, paracetamol, pantocid, furosemide, tabs sildenafil and aldactone. Arterial line was removed. On 6DPO, levosimendan and dexmedetomidine infusion were stopped. Potassium containing oral feeds was introduced. Chest X-ray revealed left lung collapse and improvised intermittent CPAP with oxygen over water was commenced with ongoing chest physiotherapy. 8DPO saw the removal of NG tube and central line. On 9DPO, Meronem, PCM and IV furosemide were stopped while tabs furosemide, oral paracetamol and oral cefuroxime were started. Oral Antimalaria (ACT) was also started. On 11DPO, the Child was discharged home conscious, alert, active and recuperating well and post operative visit scheduled for 4 weeks (Figure 3).

## Discussion

It is widely accepted that brain injury occurs after 4 minutes of circulatory arrest in normal temperature. It is also postulated that cerebral metabolism decreases by 6-7% for every 1°C decrease in temperature from 37°C, which implies that brain cooling results in a reduction in oxygen requirements. This not only protects from brain ischemia but also that of myocardium. Circulatory arrest is typically undertaken at 18-20°C and a range of safe periods for DHCA have been reported at this temperature. It has been suggested that patients can tolerate up to 30mins of DHCA without significant neurological dysfunction [1]. Longer periods of DHCA are tolerated in neonates and infants compared with adults with very low risk of neurological impairment later in childhood [2]. Despite its benefit, prolonged can result in a number of problems of coagulopathy, post ischemic hypothermia and cerebral microembolism. Profound hypothermia is associated with Dysrhythmias due to electrolyte imbalance, increased plasma viscosity and erythrocyte rigidity, metabolic acidosis, hyperglycemia and altered drug distribution and elimination [1,3]. In our own case, DHCA was done as an emergency because of loss of peripheral circulation following PDA repair which was hitherto the source of blood to the limbs as a result of the aberrant right subclavian artery- a very rare condition. It was done to both enabling the surgeon to have a clear view and locate the source of loss of circulation and then to re-attach the right subclavian artery to the ascending and descending aorta. The total duration of DHCA was 33 mins at 16°C after which the temperature was gradually brought up to 35.7°C. The child showed good recovery no neurological impairment found immediately post operatively and afterwards.

## Conclusion

DHCA as an emergency is rare and usually planned prior to surgery. In our own case, a very rare case of a congenital vascular anomaly was discovered and necessitated the use of DHCA to preserve the function. In a developing country where more sophisticated imaging is not readily available, this skill may be useful to acquire and develop for potential emergency life saving interventions.
